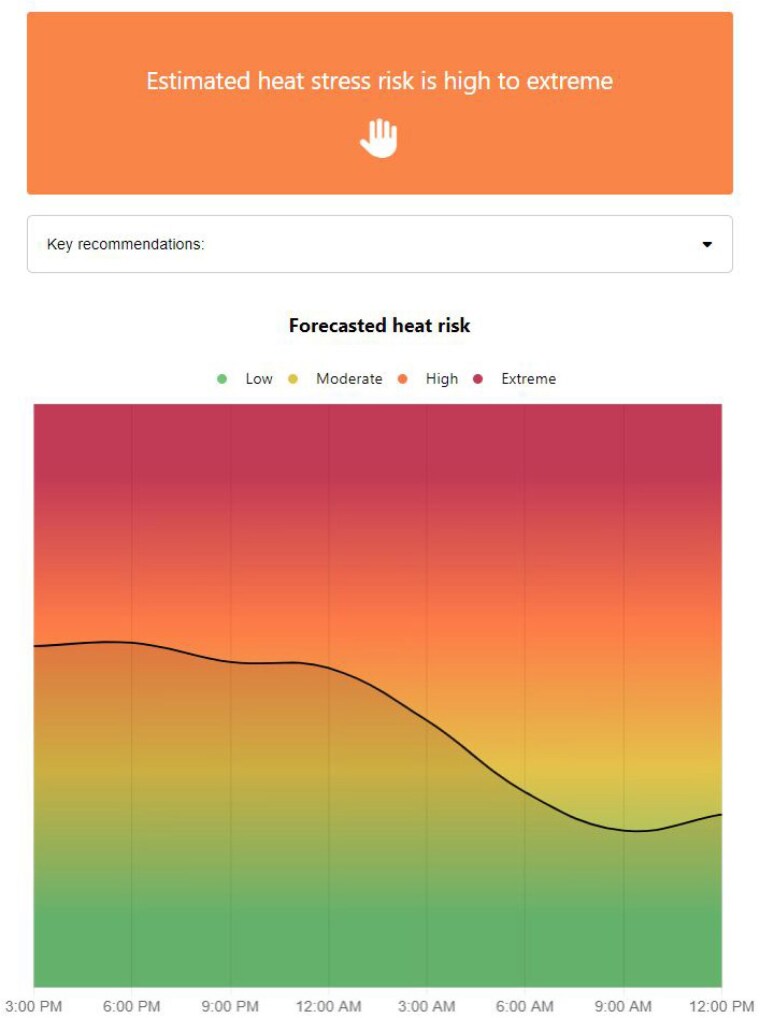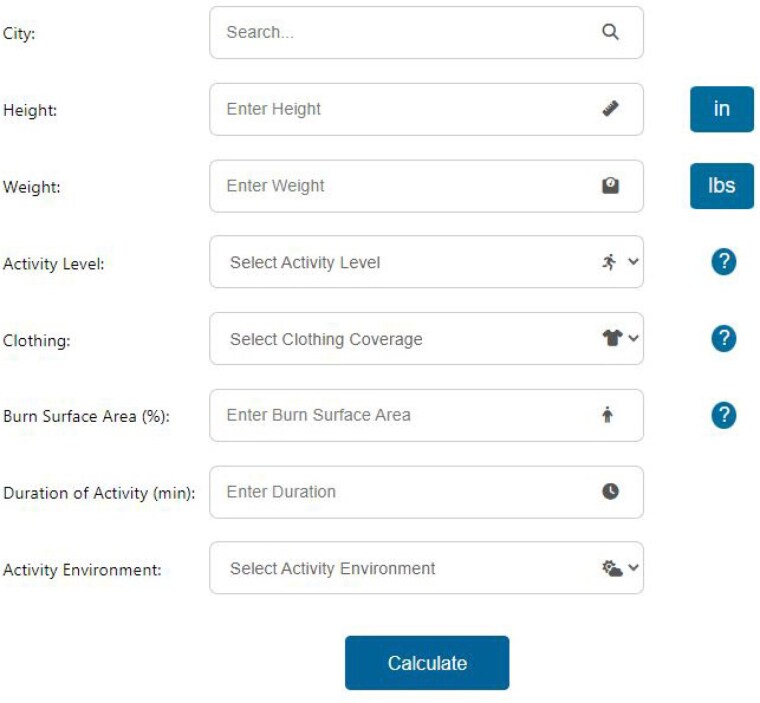# 687 A Free Online Tool to Predict Heat Risk During Physical Activity in Well-healed Burn Survivors

**DOI:** 10.1093/jbcr/iraf019.316

**Published:** 2025-04-01

**Authors:** Craig Crandall, Zachary McKenna, Josh Foster, Whitley Atkins, Elizabeth Gideon, Ollie Jay, Federico Tartarini, Erika Mii, Eric Wang

**Affiliations:** University of Texas Southwestern Medical Center; Institute for Exercise and Environmental Medicine; King’s College London; Institute for Exercise and Environmental Medicine; Institute for Exercise and Environmental Medicine; University of Sydney; University of Sydney; University of Texas at Dallas; University of Texas at Dallas

## Abstract

**Introduction:**

Burn survivors have an increased risk of heat-related illnesses and experience a heightened perceptual strain during physical activity. This is due to severely impaired body temperature regulation secondary to the absence of sweating from grafted skin. Both responses will dissuade well-healed burn survivors from receiving the cardiovascular and metabolic benefits of being physically active. We propose that informing burn survivors of their unique risk of excessive elevations in core temperature, or more importantly the lack thereof, will promote physical activity in this population.

**Methods:**

We developed a burn survivor heat risk calculator, which is a freely accessible web-based tool to inform burn survivors of their heat risk during physical activity. Gagge’s two-node thermal physiological model was modified to account for the adverse thermoregulatory consequences of burn injuries, namely a decreased ability to sweat from burn-injured skin. Model inputs include the percent body surface area burned, local environmental conditions (obtained from public domain weather data from the inputted city), physical activity intensity and duration, body height and weight, and clothing worn (Figure 1).

**Results:**

The developed and validated tool forecasts a personalized heat risk across the ensuing 20-hour period, based on predicted core temperature responses calculated from the indicated parameters (Figure 2).

**Conclusions:**

This freely available online tool is designed to be user-friendly and simple to use. The output informs the burn survivor of their unique heat risk status based on their physical characteristics, selected physical activity, and local environmental conditions.

**Applicability of Research to Practice:**

We propose that thermal intolerance contributes to burn survivors living a more sedentary lifestyle than the general population (PMID: 24043241), which contributes to a heightened incidence of cardiovascular and metabolic diseases (PMID: 26777451). This tool will convey to the burn survivor their unique heat risk of performing the desired physical activity, while reassuring each that the activity is safe when the calculator rates that heat risk level as low to moderate.

**Funding for the Study:**

This project was funded by the NIH.